# Normal left atrial diameter is associated with better performance on a cognitive screener among a cohort of ischemic stroke patients

**DOI:** 10.3389/fneur.2022.1028296

**Published:** 2022-11-24

**Authors:** Emma Gootee, Colin Stein, Alex Walker, Nicholas O. Daneshvari, Michael J. Blaha, Joao A. C. Lima, Rebecca F. Gottesman, Michelle C. Johansen

**Affiliations:** ^1^Department of Neurology, The Johns Hopkins University School of Medicine, Baltimore, MD, United States; ^2^The Johns Hopkins University School of Medicine, Baltimore, MD, United States; ^3^Department of Cardiology, The Johns Hopkins University School of Medicine, Baltimore, MD, United States; ^4^National Institute of Neurological Disorders and Stroke Intramural Research Program, Bethesda, MD, United States

**Keywords:** left atrial diameter (LAD), cognitive test batteries, stroke outcome, stroke, ejection fraction (EF)

## Abstract

**Background:**

Cardiac structure is an important determinant of ischemic stroke (IS) etiology; however, whether an association between cardiac structural markers and cognition post-IS exists is not yet established. The aim of this study is to examine the association between LAD and LVEF with cognitive performance among IS patients.

**Methods:**

IS patients admitted to the Johns Hopkins Hospital (2017–2019) underwent transthoracic echocardiography. IS was classified (TOAST) by a masked reviewer. Left atrial diameter (LAD) was evaluated as a non-linear continuous variable with one spline knot at 4 cm; left ventricle ejection fraction (LVEF) was dichotomized, then further evaluated as a non-linear continuous variable with spline knots at 50% and 70%. Patients were contacted by telephone on average 422 days post-stroke and administered the Six-Item Screener (SIS) to assess for dementia. SIS scores were dichotomized into low and high, imputing low scores for non-answerers. Multivariable logistic regression determined the association of SIS category with LAD or LVEF. A sensitivity analysis re-evaluated the association between SIS category and LAD, excluding participants with atrial fibrillation (AF).

**Results:**

Participants (*N* = 108) were on average 61 years old (range = 18–89 years), 55% male, and 63% Black. Among patients considered to have a normal LAD (≤ 4 cm), a 1 mm larger LAD was associated with 1.20 greater odds (95%CI = 1.05–1.38) of scoring in the high SIS category in the final adjustment model. This association remained significant when excluding participants with prevalent AF. There was no association between a 1 mm larger LAD and SIS category among patients with a LAD >4 cm in both the primary analysis and the sensitivity analysis. There was no association between LVEF and SIS category.

**Conclusions:**

In this prospective study, among ischemic stroke patients with a LAD within the normal range, a 1 mm increase in LAD was associated with higher scores on a telephone cognitive battery, without an association found among those with a LAD >4 cm.

## Introduction

Stroke is a leading cause of disability, with cognitive impairment impacting ~25–30% of patients after an ischemic stroke (1). The severity of the cognitive impairment that develops post-stroke is likely reflective of the characteristics of the stroke itself, such as the size or location of the infarct, as well as other pre-existing stroke risk factors (e.g., hypertension) that have also been linked to cognitive decline. Evaluating the cardiac function of patients with ischemic stroke is important in identifying the cause of stroke; but whether an association exists between the cardiac structure and function and post-stroke cognition among stroke patients is unknown, particularly if the patients do not have symptomatic cardiac disease.

The left atrium (LA) should be evaluated for the presence of thrombus in acute stroke care, as even in the absence of an acute clot incremental changes in the LA size and function have been associated with cardioembolic stroke (2, 3). For example, a higher risk of stroke exists among those with an enlarged LA compared to those with a normal sized LA, and patients with an enlarged LA are also at over three times higher risk of stroke recurrence (2, 4). Recent evidence has also shown that measures of worsening age-related LA reservoir function and stiffness are associated with higher odds of subclinical cerebral infarcts and stroke in patients with a normal ejection fraction and without atrial fibrillation (AF) (5). Apart from causing embolic stroke, tachyarrhythmias of the LA, such as AF, have been associated with a greater risk of cognitive decline and dementia. Recently, the presence of LA enlargement (left atrial volume index ≥34 mL/m^2^) and prevalent AF were found to be associated with a decline in cognitive functioning over time, but not with LA enlargement alone (6).

Abnormal left ventricular ejection fraction (LVEF), a measure of left ventricular systolic function, is also a known risk factor for ischemic stroke. A recent study examining the association between left ventricular (LV) function and risk of recurrent stroke found that, for every 5 percentage points decrease in LVEF, even within the normative range, there was an 18% increase in the risk of stroke (7). LV function has also been found to be associated with cognition, with the presence of impaired cognitive function in those with heart failure (HF) being as high as 53% (8). While HF and AF are known to be associated with cognitive impairment, whether abnormalities in the structure and function of the LA or LV among patients without symptomatic disease are associated with cognition post-stroke is unknown. Additionally, although there has been suggestion that poorer LV systolic function on echocardiography is associated with smaller brain volumes, whether abnormalities in LVEF are associated with poorer results on cognitive batteries is unknown (9). Furthermore, limited research has evaluated the association between LVEF or LAD with cognitive performance in a cohort of ischemic stroke patients.

The overarching goal of this study is therefore to examine the association between LAD and LVEF with cognitive performance in a cohort of ischemic stroke patients. We hypothesize that participants with a normal LVEF will score better on the cognitive assessment than those with a decreased or hyperdynamic LVEF, and that participants with a LAD ≤ 4 cm will score better than those with an enlarged LAD.

## Methods

### Study population

**Figure 1:**
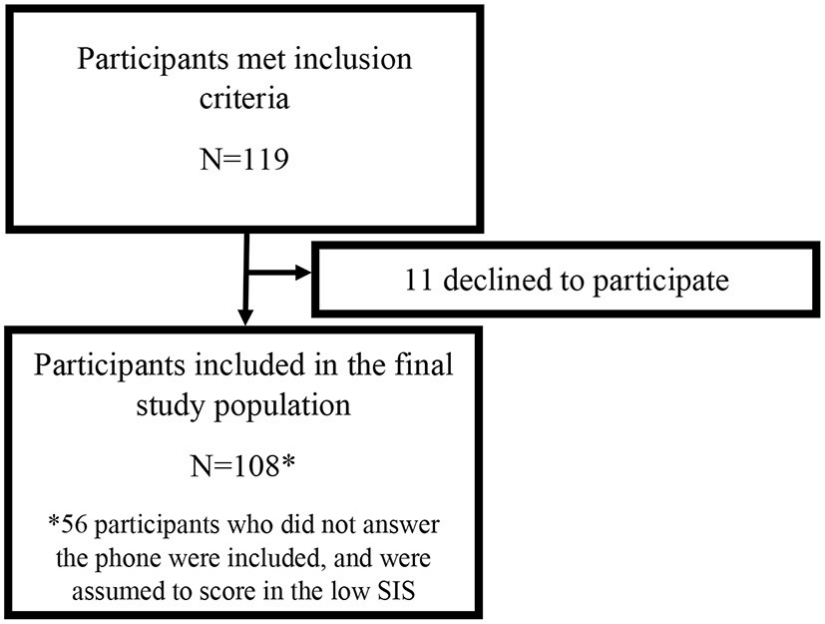
Participant flow diagram.

Participants for this study were recruited as part of an ongoing prospective study of ischemic stroke patients at the Johns Hopkins Hospital (JHH), a comprehensive tertiary referral center for stroke. This study was approved by the JHH Institutional Review Board, and all participants gave written informed consent. Patients were approached for consent if they were admitted to the JHH between 2017 and 2019 with an acute ischemic stroke confirmed on cerebral magnetic resonance imaging (MRI), were adults at least 21 years of age, did not have a documented history of dementia, had capacity to provide informed consent for themselves, and had a clinical indication for a transthoracic echocardiogram (TTE), which at JHH is standard imaging obtained in stroke patients upon admission. Previously enrolled participants were contacted by phone on average 422 days post-stroke (SD = 229), and verbal consent was obtained to perform the Six-Item Screener (SIS, Figure 1). As this is an ongoing prospective study, participants will continue to be followed for up to 5 years post-consent. Participants with stroke lesion volumes >150 mL were excluded since they represent severe outliers and participants with extremely large stroke volumes would likely have severe cognitive impairment. Demographics and vascular risk factors were collected at the time of initial consent.

### Echocardiography assessment

At JHH, a TTE is routinely obtained in ischemic stroke inpatients for a cardiovascular evaluation, with the decision to order a TTE at the discretion of the inpatient treatment team, although it is part of the admission order set. Two-dimensional echocardiography is performed by certified sonographers following standardized protocol based on the American Society of Echocardiography guidelines (10). Patients are examined in a left lateral decubitus position, utilizing parasternal long axis, parasternal short axis, apical 2-chamber, apical 3-chamber, and apical 4-chamber traditional views. Electrocardiogram (ECG) tracing is recorded during the examination. All recording and measurements are done at the end-expiration, with a full 3-cardiac cycle capture. LVEF was calculated by cardiologists at the time of the reading of the TTE (%) and the LAD was measured in the anterior-posterior direction (cm) from the parasternal long axis view.

### Telephone six-item screener

The SIS is a brief and reliable instrument that assesses global cognitive function by asking three orientation and three recall questions (11). This instrument can be used to identify cognitive impairment in patients with a sensitivity of 97.7% and has diagnostic properties comparable to the full mini-mental state examination (MMSE) (11). The test is scored 0–6, with a point given for each correct answer and thus higher scores indicating better cognition.

Two research assistants with training and expertise in cognitive batteries contacted the participants by phone after they were discharged from the hospital to obtain informed consent to administer the SIS. Participants were contacted by telephone up to three times before they were marked as a “non-answerer.” If another person responded to the phone call, the participant was re-contacted at a time that they were said to be available. For participants who gave consent, the medical history was first updated to capture recurrent stroke events or new diagnosis of AF. The operational procedure for administering the SIS was that participants were first given the three words to remember (grass, paper, shoe) and then asked three orientation questions (the year, the month, and the day of the week). They were instructed not to write down the recall words, and not to ask for assistance during the test. After they answered the orientation questions, they were then asked to recall the three words they had been given in the beginning. Participants were given one attempt to answer questions, but self-corrections were permittable (e.g., if they incorrectly recalled glass and then immediately corrected their answer to correctly recall grass, the question was marked as correct). Testing lasted, on average, 10 min, and no participants requested more time.

### Stroke infarct volume calculation

All participants underwent a clinically indicated 3-T brain MRI with contrast. Sequences included were Axial T1, Axial Diffusion-Weighted Image (DWI), Axial T2^*^ GRE, and Axial T2 Flair. Infarct volumes were calculated by a reviewer masked to patient characteristics and stroke subtypes who manually traced hyperintense areas of diffusion restriction on T2 DWIs using MRIcron© software. 30% of MRI traces were repeated by another reviewer to determine interrater reliability, which was >90%. All scans were normalized to a reference scan to standardize voxel size, number of axial slices, and resolution. Volumes were calculated using Statistical Parametric Mapping 12 (SPM12; Wellcome Center for Human Neuroimaging, London, UK), which operates using MATLAB R2007a (7.4) to R2019b (9.7) (Mathworks, Natick, MA, USA). For the purpose of analysis, infarct volumes were converted from mm^3^ to milliliters (mL), and a 5 mL increment was used when reporting results.

### Statistical analysis

The primary dependent variable was the SIS, and the primary independent variables were, in separate analyses, LAD (mm) and LVEF (%). Scores on the SIS were dichotomized for analysis purposes into a “low” category (0–3 points, worse score) and “high” category (4–6 points, better score). LAD was evaluated as a non-linear continuous variable with a spline knot at 4 cm. LAD was considered to be normal in diameter if ≤ 4 cm or enlarged if the diameter was >4 cm, as suggested by established echocardiography guidelines (12). LVEF was first dichotomized into two categories, one to represent abnormal LVEF ( ≤ 50 and ≥70%) and another to represent normal LVEF (50–70%). LVEF was then further evaluated as a non-linear continuous variable with spline knots at 50 and 70%. In this manuscript, LVEF ≤ 50% will be referred to as decreased function, 50–70% will represent normal function, and >70% will be referred to as hyperdynamic function, as suggested by American College of Cardiology guidelines (13).

Multivariable logistic regression models were constructed to determine the association between SIS category and LAD or LVEF, each in separate models, adjusted for potential confounders in stepwise models. When evaluated as non-linear continuous variables with spline knots, the effect estimates of these analyses would be interpreted as the change in SIS per unit change in the cardiac variable (per 1 mm change in LAD, 1% change in LVEF), within the bin of the variable. Covariates were collected and included in the adjustment models if they were believed to strongly confound the relationship between cardiac variables and SIS score. Model 1 adjusted for age, self-reported race (Black vs. other), and sex; Model 2 additionally adjusted for ischemic stroke infarct volume as previously detailed (mL); Model 3 additionally adjusted for history of hypertension. Hypertension was defined as either a documented history of hypertension in the medical record, taking hypertension medications, or reported by the patient at the time of consent. Scores of participants who did not answer the phone (*N* = 56) were imputed into the low SIS group, rather than excluding them from analysis. Given that there is a potential that AF might be on the causal pathway between LAD and post-stroke cognition, a sensitivity analysis was performed in which participants with prevalent AF at the time of admission were excluded. All statistical analyses were performed using Stata v17.0 (14). Two-sided *P* < 0.05 was considered statistically significant.

### Standard protocol approvals, registrations, and patient consents

This study has been approved by the Johns Hopkins Medicine Institutional Review Board and all patients provided informed consent.

## Results

### Study population

A total of 119 participants met inclusion criteria for this study (Figure 1), 11 of which declined to participate. Of the remaining 108, 52 provided consent and 56 never responded. Participants were predominantly male (55%) and Black individuals (63%) with a mean age of 61 years old (SD = 13.36). Sixty-five participants scored in the low SIS group, and 43 scored in the high SIS group. The mean SIS score among those in the low category was 2.58 (SD = 0.90) and among those in the high category was 4.88 (SD = 0.79). Cardioembolic stroke occurred in 11 participants (17%) who scored in the low SIS category and 12 participants (28%) who scored in the high SIS category, and there was no difference in prevalence of cardioembolic stroke between the high and low SIS groups (*p* = 0.17). In the low SIS category, the cause of stroke was large artery atherosclerosis for 13 participants (20%), small vessel disease for 15 participants (23%), other etiology for 6 participants (9%), and undetermined etiology (cryptogenic) for 20 participants (31%). In the high SIS category, the cause of stroke was large artery atherosclerosis for 6 participants (14%), small vessel disease for 14 participants (33%), other etiology for 2 participants (5%), and undetermined etiology (cryptogenic) for 9 participants (21%). There were no statistically significant differences in stroke subtype between the low and high SIS categories. Participants in the high and low SIS categories did not significantly differ in demographics or vascular risk factors aside from BMI (Table 1), with participants in the high SIS category (mean = 31.91 kg/m^2^, SD = 8.10) having significantly higher BMIs (*p* = 0.005) than those in the low SIS category (mean = 27.84 kg/m^2^, SD = 6.58).

**Table 1:** Ischemic stroke patient demographic information.

**Variable**	**Low SIS (0–3)** ** (*n* = 65)**	**High SIS (4–6)** ** (*n* = 43)**	***p*-value**
Age [mean (SD)]	60.0 (13.5)	59.0 (14.3)	0.71
Female sex	27 (42%)	18 (42%)	0.97
Black race	40 (62%)	27 (63%)	0.90
NIHSS [mean (SD)]	3.9 (4.9)	4.1 (4.5)	0.84
History of hypertension^*^	50 (77%)	34 (79%)	0.79
History of diabetes^*^	22 (34%)	18 (42%)	0.40
History of atrial fibrillation^*^	9 (14%)	8 (19%)	0.51
BMI (kg/m^2^) [mean (SD)]	**27.8 (6.6)**	**31.9 (8.1)**	**0.005**
LDL cholesterol^*^ [mean (SD)]	**109 (45.4)**	**98 (40.4)**	**0.20**
Stroke lesion volume (cm^3^) [mean (SD)]	**15.3 (22.8)**	**8.9 (14.4)**	**0.11**
Tobacco use^*^			**0.71**
Never	**27 (42%)**	**15 (35%)**	
Former	**21 (32%)**	**17 (40%)**	
Current	**17 (26%)**	**11 (26%)**	

Results are N (%) unless otherwise specified.

SIS, six-item screener; NIHSS, NIH stroke scale; BMI, body mass index.

Bolding indicates significant finding (*p* < 0.05).

* History of hypertension, diabetes, atrial fibrillation, and tobacco defined as history of disease/behavior at time of initial stroke admission. LDL cholesterol collected from admission lab work.

### Associations of LA diameter and post-stroke cognition

**Figure 2:**
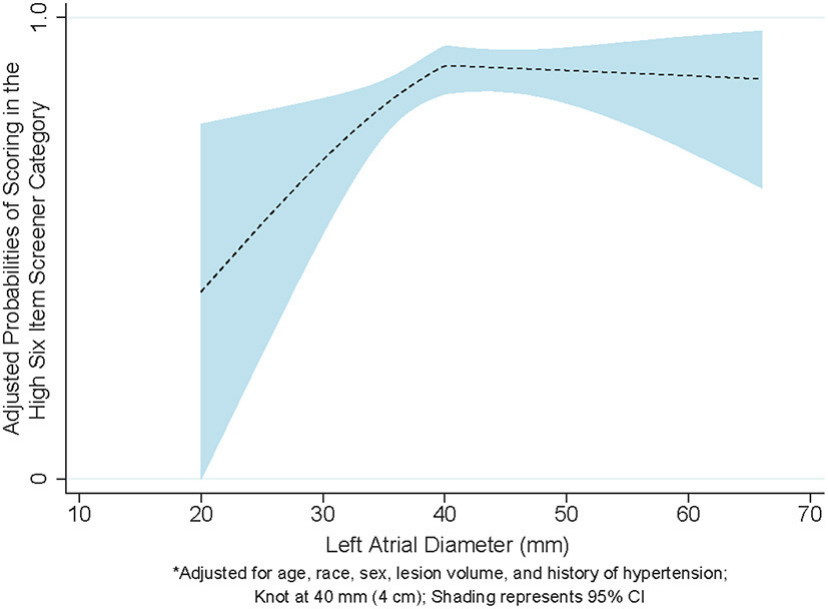
Linear spline model demonstrating the association between left atrial diameter and six-item screener.

Of 108 participants, 66 had a LAD ≤ 4 cm and 42 had a LAD >4 cm. The mean LAD was 3.93 cm (SD = 0.80). There was not a significant difference in mean LAD between those scoring in the high SIS category and those scoring in the low SIS category (*p* = 0.12). Among patients with a LAD within the normal range (0–4 cm), a 1 mm larger LAD was significantly associated with 1.20 times higher odds of scoring in the high SIS category in the fully adjusted model (95% CI = 1.05–1.38; Figure 2). Among patients with a LAD outside of the normal range (>4 cm), each 1 mm larger LAD was not significantly associated with performance on the SIS (Model 3: OR = 0.99, 95% CI = 0.91–1.07).

### Associations of LA diameter and post-stroke cognition when excluding participants with atrial fibrillation

A sensitivity analysis removed 17 participants with prevalent AF at the time of initial consent. Among the participants without AF (*N* = 91), a 1 mm larger LAD among those with LAD ≤ 4 cm was associated with a significantly higher odds of scoring in the high SIS category in the fully adjusted model, with a similar effect estimate to that of the primary result (OR = 1.19, 95% CI = 1.02–1.37). This association remained significant across all three adjustment models (Table 2). As before, there was no association between a 1 mm larger LAD and SIS scores among those with a LAD >4 cm (Model 3: OR = 1.01, 95% CI = 0.90–1.14).

**Table 2:** Logistic regression analysis demonstrating the odds of scoring in the high SIS category per unit change within the specified LAD range, among ischemic stroke survivors without AF.

**Left atrial diameter (per 1 mm change; *n* = 91)**	**Model 1** ^*^		**Model 2**		**Model 3**	
	**OR**	**95% CI**	**OR**	**95% CI**	**OR**	**95% CI**
0–4 cm “normal”(range)	**1.17**	**1.01**–**1.36**	**1.18**	**1.02**–**1.37**	**1.19**	**1.02**–**1.37**
>4 cm “abnormal”(range)	1.02	0.91–1.15	1.01	0.90–1.14	1.01	0.90–1.14

TTE, transthoracic echocardiogram; LAD, left atrial diameter; OR, odds ratio; CI, confidence interval; SIS, six-item screener; AF, atrial fibrillation.

* Adjustment Models: Model 1: Age, Race, Sex; Model 2: Model 1 + Ischemic Stroke Infarct Volume (cm^3^), Model 3: Model 2 + History of hypertension.

Bolding indicates statistically significant finding (*p* < 0.05).

### Associations of LVEF and post-stroke cognition

LVEF ranged from 17 to 85%, with a mean LVEF of 62% (SD = 10.9). When evaluated as a dichotomous variable, 75 participants had a LVEF between 50 and 70%, and 33 had a LVEF outside of these confines ( ≤ 50 and ≥70%). When evaluated as a non-linear continuous variable with two spline knots, 12 participants had LVEFs ≤ 50%, 75 were within the 50–70% range, and 21 had LVEFs >70%. There was no significant association between LVEF and performance on the SIS when considered either as a dichotomous variable (Model 3: OR = 1.32, 95% CI = 0.55–13.15) or when considered as a non-linear continuous variable (Table 3). These results remained insignificant across all adjustment models for potential confounders.

**Table 3:** Logistic regression analysis demonstrating the odds of scoring in the high SIS category per unit change within the specified LVEF range among stroke survivors.

**Left ventricular ejection fraction (per 1% change;** ***n* = 108)**	**Model 1** ^*^		**Model 2**		**Model 3**	
	**OR**	**95% CI**	**OR**	**95% CI**	**OR**	**95% CI**
≤ 50%“decreased”	0.97	0.88–1.07	0.96	0.87–1.07	0.96	0.86–1.06
50–70%“normal”	0.97	0.90–1.04	0.97	0.90–1.04	0.97	0.90–1.05
>70%“hyperdynamic”	1.07	0.88–1.31	1.10	0.90–1.35	1.09	0.89–1.34

TTE, transthoracic echocardiogram; LVEF, left ventricular ejection fraction; OR, odds ratio; CI, confidence interval; SIS, six-item screener.

* Adjustment Models: Model 1: Age, Race, Sex; Model 2: Model 1 + Ischemic Stroke Infarct Volume (cm^3^), Model 3: Model 2 + Patient history of hypertension.

## Discussion

Among a prospective cohort of ischemic stroke patients, we found that an incrementally larger LAD was significantly associated with an increased likelihood of scoring higher on a post-stroke cognitive assessment, administered on average 14 months after the stroke event. This association was only observed among stroke participants with a LAD ≤ 4 cm, which is considered in the normal range of LA size. We did not find an association between LAD and scores on the SIS among stroke patients whose LAD was enlarged (i.e., > 4 cm), although one might anticipate that those with an enlarged LAD would score worse on the SIS.

Prior research has found that as many as 38% of ischemic strokes result in cognitive impairment, with variation among ischemic stroke subtypes (15). Data from several studies yielded estimates that as many as 1-in-10 stroke patients developed new dementia after a first stroke, and over 1-in-3 were demented after a recurrent stroke (15–17). As the risk of death from ischemic stroke has declined, the number of stroke survivors, and therefore stroke patients living with cognitive impairment, has increased (18). In community-based studies with adjustment for age, the prevalence of dementia in people with a history of stroke is ~4–6 times higher than in those without a history of stroke (19). The Helsinki study reported that up to 83% of stroke survivors show impairment in at least one cognitive domain, and 50% show impairment in multiple cognitive domains (20). Due to this burden, it is imperative that the underlying mechanisms driving these associations are well-understood.

AF, which can lead to an enlarged LA, is associated with poorer cognitive functioning compared to those without AF, with two important mechanisms being both clinically apparent ischemic stroke and silent brain infarction (21). There is an increased recognition of the importance of the structure and function of the LA in stroke etiology, apart from simply being a sign of AF, so it is interesting to also consider how the LA may modulate the cognitive trajectory of patients post-stroke. A large, community-based prospective cohort study of older adults found that participants with LA enlargement had lower executive and global cognitive function than participants with normal LA size, independent of AF (6). A smaller, community-based cohort determined that LAD is independently associated with cognitive function based on their finding that participants with larger LADs did poorer on language, delayed memory, and total index composites of the Repeatable Battery for the Assessment of Neuropsychological Status (RBANS) (22, 23). Of note, neither study included a cohort of ischemic stroke patients (6, 23).

We found that, in a cohort of ischemic stroke patients, participants were more likely to have a higher, or better, score on the SIS with each 1 mm larger LAD, but only within the normal range of LA size. Beyond the normal range, there was no association between an incrementally larger LAD and performance on the SIS. One might anticipate, as we had hypothesized, that there would be an inverse association with an incrementally larger LAD being associated with worse performance on the SIS among participants with an LA that is abnormal or enlarged. We did not find this association, but it may be that we were underpowered to detect a difference, or that the follow-up duration was not long enough to pick up the anticipated poorer cognition. It may also be that patients with more severe LA function, or very large LAD, were less likely to be alive, or without recurrent stroke, at the time that we contacted them. As a result, the patients who answered the phone would be more likely to have a better score as a result of having a better outcome. In modeling the data, there was a clear inflection point at 4 cm, suggesting that ischemic stroke patients with a normal-sized LA were different than those with a LA that was enlarged, although the exact differences cannot be definitively determined from our results.

This study found no association between LVEF and score on a cognitive assessment among patients with ischemic stroke. Prior research has suggested that abnormal cognitive changes, as measured by the Boston Naming Test, only occur within participants in the lowest and highest quintiles of LVEF (24). There have also been previous studies that suggested that poorer LV systolic function on echocardiography is associated with smaller brain volumes (9). This association was not reflected in the present study; however, we are limited by a small sample size, which could offer one explanation as to why these findings were not replicated or the relationship may be more nuanced than we were able to capture.

This study is novel in that it explores the relationship between cardiac structures and function with cognition specifically in a cohort of ischemic stroke patients. However, we recognize that there are some limitations to our study. The assumption that participants who did not answer the phone would score in the low SIS category may not have been a fair assumption. This assumption was made as it is the more conservative approach; however, there are other factors that could have led someone to not answer their phone, like working outside of the home, that may actually be associated with higher cognitive performance. It could also be that we did not wait long enough post-stroke to capture stroke-associated cognitive impairment, as participants were administered the SIS on average 13 months post-stroke. The SIS was developed as a brief screen for cognitive impairment that aims to balance diagnostic accuracy with brevity. It has a number of strengths, including that each of the six items on the SIS comes from the MMSE, which allows for comparison among studies utilizing either test. The sensitivity and specificity of the SIS are comparable to those of the full MMSE, though these vary when the number of errors used as a cut-off point are adjusted. While it is an excellent screening tool, the SIS is limited in the domains that it tests and could potentially overlook deficits in other domains. Finally, this analysis represents a cross-sectional analysis of a larger, ongoing prospective study that did not obtain cognitive measures at baseline, and therefore we were not able to determine change in cognition over time. Follow-up however is ongoing, and we anticipate being able to expand this analysis in the future.

## Conclusion

Stroke is a leading cause of disability which results in cognitive impairment in many patients (1). Due to this risk, it is important not only to understand how stroke impacts cognition but also which, if any, risk factors for stroke are associated with cognition. We cautiously suggest that the results of this study may imply that the size and function of the left atrium could be important in the degree of cognitive impairment post-stroke. Further studies should consider cardiac structure and function when evaluating cognition in ischemic stroke patients.

## Data availability statement

Anonymized data not published in this article will be shared upon reasonable request to any qualified investigator.

## Ethics statement

The studies involving human participants were reviewed and approved by Johns Hopkins School of Medicine. The patients/participants provided their written informed consent to participate in this study.

## Author contributions

EG performed statistical analysis and wrote the manuscript. CS, AW, and ND collected data and reviewed the manuscript. MB, JL, and RG reviewed the manuscript and evaluated the intellectual content of the manuscript. MJ designed the study, performed statistical analysis, reviewed the manuscript, and evaluated intellectual content of the manuscript. All authors contributed to the article and approved the submitted version.

## Funding

MJ receives funding through the American Heart Association Career Development Award (#19CDA34660295) and the National Institute of Neurological Disorders and Stroke (#K23NS112459). RG was supported by the National Institute of Neurological Disorders and Stroke Intramural Research Program.

## Conflict of interest

The authors declare that the research was conducted in the absence of any commercial or financial relationships that could be construed as a potential conflict of interest.

## Publisher's note

All claims expressed in this article are solely those of the authors and do not necessarily represent those of their affiliated organizations, or those of the publisher, the editors and the reviewers. Any product that may be evaluated in this article, or claim that may be made by its manufacturer, is not guaranteed or endorsed by the publisher.
